# Prevalence of Delta-Like Protein 3 in a Consecutive Series of Surgically Resected Lung Neuroendocrine Neoplasms

**DOI:** 10.3389/fonc.2021.729765

**Published:** 2021-09-09

**Authors:** Greta Alì, Iosè Di Stefano, Anello Marcello Poma, Stefano Ricci, Agnese Proietti, Federico Davini, Marco Lucchi, Franca Melfi, Gabriella Fontanini

**Affiliations:** ^1^Unit of Pathological Anatomy, University Hospital of Pisa, Pisa, Italy; ^2^Department of Surgical, Medical, Molecular Pathology and Critical Area, University of Pisa, Pisa, Italy; ^3^Pathology Unit, Azienda Unità Sanitaria Locale-IRCCS di Reggio Emilia, Reggio Emilia, Italy; ^4^Multispecialty Centre for Surgery, Minimally Invasive and Robotic Thoracic Surgery, University Hospital of Pisa, Pisa, Italy; ^5^Unit of Thoracic Surgery, University Hospital of Pisa, Pisa, Italy

**Keywords:** lung neuroendocrine tumors, delta-like protein 3, immunohistochemistry, prognosis, biomarker

## Abstract

Delta-like protein 3 (DLL3) is a protein of the Notch pathway, and it is a potential therapeutic target for high-grade lung neuroendocrine tumors (NETs), i.e., small cell lung carcinoma (SCLC) and large cell neuroendocrine carcinoma (LCNEC). However, DLL3 prevalence in lung NETs and its association with clinicopathological characteristics and prognosis remained unclear. We analyzed the immunohistochemical expression of DLL3 and its prognostic role in a consecutive series of 155 surgically resected lung NETs, including typical carcinoid (TC), atypical carcinoid (AC), LCNEC, and SCLC patients. The DLL3 expression was categorized as high (>50% positive tumor cells) or low (<50%). In addition, tumors were categorized by H-score (i.e., percentage of positive cells by staining intensity, ≥150 *vs.* <150). DLL3 staining was positive in 99/155 (64%) samples, and high DLL3 expression was frequently observed in high-grade tumors. In detail, 46.9% and 75% of SCLC and 48.8% and 53.7% of LCNEC specimens showed a high DLL3 expression by using H-score and percentage of positive tumor cells, respectively. Regarding low-grade NETs, only 4.9% and 12.2% TCs and 19.5% and 24.4% ACs had high DLL3 expression considering H-score and percentage of positive tumor cells, respectively. High DLL3 expression was associated with advanced American Joint Committee on Cancer (AJCC) stage, peripheral location, and chromogranin A expression in high-grade tumors (p < 0.05). In low-grade NETs, high DLL3 expression was associated with female sex, peripheral location, a higher number of mitoses, higher Ki-67 index, presence of necrosis, and pleural infiltration (p < 0.05). No association was observed between high DLL3 expression and overall survival (OS) and disease-free survival (DFS) in high-grade NETs, whereas high DLL3 expression was associated with lower DFS in ACs (p = 0.01). In conclusion, our study demonstrated a high prevalence of DLL3 expression in high-grade lung NET patients and its association with aggressive clinicopathological features. These findings confirm that DLL3 could represent a useful biomarker for target therapy in high-grade tumors. Our results also suggest that the DLL3 expression could identify a subset of AC tumors with more aggressive behavior, thus providing the basis for new therapeutic options in this group of patients.

## Introduction

Neuroendocrine (NE) tumors (NETs) are a heterogeneous group of neoplasms found most commonly in the lung and in the gastrointestinal tract (1, 2). The 2021 WHO classification of lung tumors identifies four distinct histological variants of lung NETs by using diagnostic criteria similar to those used since the 1999 WHO classification. These lung NETs have been categorized as typical carcinoid (TC), atypical carcinoid (AC), large cell NE carcinoma (LCNEC), and small cell lung carcinoma (SCLC); and they are differentiated on the basis of mitotic rate, presence of necrosis, and cytomorphological details, which allow to distinguish between low-grade (TC and AC) and high-grade (LCNEC and SCLC) tumors (3–5).

Low-grade NETs of the lung have a favorable prognosis compared with the more common high-grade NETs, i.e., LCNECs and SCLCs (6, 7).

The correct classification of lung NETs allows to select the most effective treatment regimen; surgery is often curative for both TC and AC, and for LCNECs. On the contrary, surgery is rarely used for SCLC patients, who are generally treated with chemotherapy. However, the rapid acquisition of chemoresistance in these patients and the substantial lack of alternative treatment options contribute to clinical failures (8–10). In carcinoid patients with metastatic disease, adjuvant therapy should be considered only in selected cases, since no studies have convincingly proved a benefit in terms of risk of local or distant recurrence (11–13). Therefore, more effective therapies and predictive biomarkers are needed both in carcinoid tumor patients who are not curable with surgery alone and in high-grade pulmonary NE carcinoma patients.

Delta-like protein 3 (DLL3), a member of the Notch family, has been identified as an inhibitory ligand of the Notch signalling pathway. DLL3 might function as an oncogenic driver in high-grade NETs, not only in the lung (14) but also in the gastrointestinal area (15), where DLL3 appears to be a downstream transcriptional target of the Achaete-scute homolog 1 (ASCL1) transcription factor (16–20). In particular, DLL3 is frequently expressed in the cell membrane of high-grade NETs, and it has low to no expression in most normal tissue (21); therefore, DLL3 could represent a potential therapeutic target in these tumors. Recently, some preclinical and clinical studies have used rovalpituzumab tesirine (Rova-T), a humanized monoclonal antibody against DLL3 in SCLCs (22–24). In these studies, the DLL3 expression seemed to identify patients who are more likely to achieve a response and a better long-term benefit after treatment with Rova-T (23, 24). Other DLL3-targeting agents, such as T cell-redirecting therapies and immuno-oncology therapies (AMG 757 and AMG 119), may have a high effect and specificity for DLL3-positive SCLC tumor cells (25–27).

For these reasons, recent studies have focused on the immunohistochemical DLL3 expression in lung NETs. However, most of them concern high-grade neoplasms, whereas few data are available for carcinoid tumors (23, 24, 28–32). In this study, we analyzed the immunohistochemical expression of DLL3 in a cohort of 155 patients with lung NETs including TCs, ACs, SCLCs, and LCNECs. This cohort included only limited-stage lung NETs treated with surgery; and for all cases, clinicopathological characteristics and prognostic factors were retrospectively reviewed. The aim of this paper was to investigate clinical features that might be associated with the DLL3 expression and to explore the prognostic role of this marker in pulmonary NETs.

## Materials and Methods

### Patient Selection

This study was approved by the Ethics Committee “Comitato Etico di Area Vasta Nord Ovest” (CEAVNO) for Clinical Experimentation. A total of 155 lung NET specimens were retrospectively collected from the archives of the Operative Unit of Pathological Anatomy III of the University Hospital of Pisa. In detail, we collected 41 TC, 41 AC, 41 LCNEC, and 32 SCLC samples obtained from patients who had been submitted to surgical resection at the Unit of Thoracic Surgery of the University Hospital of Pisa from December 2007 to December 2019. Participation in this study required informed consent. Patients did not receive neoadjuvant chemotherapy nor radiation therapy. Clinical information, including sex, age, smoking status, disease-free survival (DFS), and overall survival (OS) were reviewed for each patient.

### Lung Tissue Specimens

All tumor samples were formalin-fixed and paraffin-embedded (FFPE). The most representative paraffin block of tumor was selected for immunohistochemical analysis for each case. Histological diagnoses and pathological features were obtained by two pathologists (GA and ID), according to the WHO 2015 histological and immunohistochemical criteria (4).

In detail, the NET specimens were evaluated for growth patterns (organoid, trabecular, follicular, palisading, rosette, spindle-cell, and diffuse lymphoma-like), mitosis number per 2 mm^2^, presence of necrosis and its pattern (absent, punctate, extensive, and geographic), vascular invasion (none, present focal, present extended), and tumor-infiltrating lymphocytes (TILs), both intra-tumoral and stromal lymphocytes (none <1%, focal <10%, moderate <50%, and diffused ≥50%). In detail, the presence of necrosis was determined by semiquantitative analysis evaluating necrosis percentage in the tumor area. We also evaluated the immunohistochemical expression of the NE markers (chromogranin A, synaptophysin, and CD56). At least one positive NE marker was required for diagnosis. Furthermore, the immunohistochemical results for thyroid transcription factor 1 (TTF1) and Ki-67 proliferative index were available for all samples. The NE markers, Ki-67, and TTF1 were scored as negative or positive (negative, weakly 1+, moderately 2+, or strongly 3+), as described before (33). Ki-67 was evaluated as the percentage of positively stained tumor cell-nuclei.

For each tumor sample, data concerning site, size, lymph node (LN) status, pleural involvement, and stage were also collected. For all lung NETs, the eighth edition of the TNM classification was applied for pathological staging (34).

DFS was calculated from the date of tumor resection and diagnosis to the date of either disease recurrence including local recurrence or metastasis; otherwise, data were censored at the time of last follow-up or death. OS was calculated from the date of tumor resection to the date of death, or data were censored at the last follow-up.

### Immunohistochemistry

DLL3 immunohistochemical analysis was performed on 4-µm-thick tissue sections that were deparaffinized in xylene and rehydrated using a graded series of ethanol solutions. The sections were then subjected to immunohistochemical staining with anti-DLL3 antibody, Rabbit Monoclonal Primary Antibody (clone SP347) (Ventana Medical Systems, Inc. Tucson, AZ, USA) by incubating the sections at 36°C for 32 min. Analysis was conducted with the BenchMark ULTRA semiautomated staining instrument (Ventana Medical Systems) using the OptiView DAB IHC Detection Kit (Ventana Medical Systems). Following a series of washes, the sections were counterstained with Hematoxylin II for 4 min and with Bluing Reagent (Ventana Medical Systems) for 4 min, dehydrated by passages in ethanol with increasing concentration from 70% to 100%, and then mounted.

**Figure 1:**
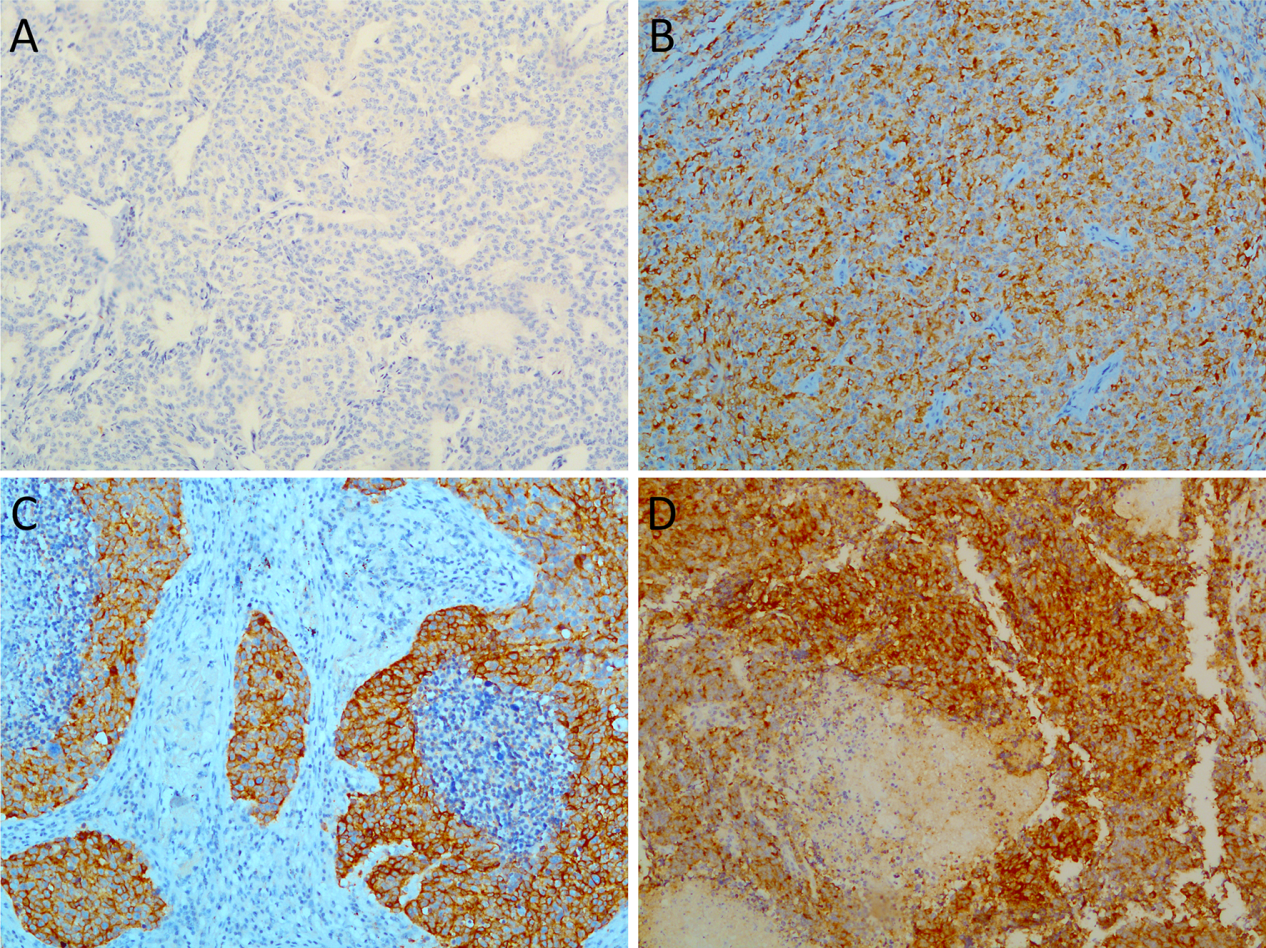
Representative images showing variable percentages of delta-like protein 3 (DLL3) immunohistochemical staining in lung neuroendocrine tumors: **(A)** typical carcinoid DLL3 negative; **(B)** a case of atypical carcinoid showing combined cytoplasmic and membranous staining with moderate intensity; **(C)** large cell neuroendocrine carcinoma with strong and diffuse DLL3 staining; **(D)** high immunohistochemical expression level of DLL3 in a small cell lung carcinoma specimen. Magnification, ×20.

In all cases, immunohistochemical evaluation was performed independently by two pathologists (GA and ID) who were blinded to all the clinical and pathological data. Selected cases were discussed with a third pathologist (GF) for confirmation. In our study, DLL3 expression was scored for any cytoplasmic and/or membranous staining at any intensity in total tumor cells. In literature, the evaluation of DLL3 immunohistochemical expression has widely varied by using different scores and thresholds for defining positivity (21–23, 28–30, 32, 35). Therefore, in our study, DLL3 positivity was determined based on the proportion of cells expressing DLL3 out of the total number of cells defining the level of expression of DLL3. Subsequently, we categorized DLL3 staining by using the following threshold for DLL3 scoring, which is used in the first clinical trial (23): high expression (>50% positive tumor cells) or low expression (<50%). We also determined the staining intensity as weak (1), intermediate (2), and strong (3) for each sample (**Figure 1**). Therefore, in order to determine the best evaluation system for DLL3 expression and to take in account the different intensities of the staining we observed in our samples, we further evaluated the DLL3 immunohistochemical results by a semiquantitative approach used to assign an H-score to tumor samples. H-score was calculated by multiplying the percentage of positive cells by the predominant staining intensity, with 300 possible values (0–300), as previously described (22, 29). As well as for the score based on the proportion of DLL3 positive cells, the tumors were ranked according to the median theoretical value as high DLL3 expressors (H-score ≥150) and low DLL3 expressors (H-score <150) by using H-score.

### Statistical Analysis

Pearson’s chi-squared test or Fisher’s exact test was used for categorical variables. Continuous variables were analyzed by the Mann–Whitney or the Kruskal–Wallis tests, and by the Dunn test for multiple comparisons with the Benjamini–Hochberg correction. Survival curves were computed by the Kaplan–Meier method. Cox’s proportional hazard model was used for both univariate and multivariate analyses. All analyses were performed in R environment (version 4.0.2, https://www.r-project.org/, last accessed in January 2021).

A p-value below 0.05 was considered significant.

## Results

### Clinicopathological Characteristics of Patients

The present study included 155 patients with lung NETs, 41 (26.5%) TCs, 41 (26.5%) ACs, 41 (26.5%) LCNECs, and 32 (20.5%) SCLCs. Clinicopathological characteristics of patients and morphological findings are summarized in **Table 1**.

**Table 1:** Clinicopathological characteristics of patients with lung neuroendocrine tumors.

Features	All patients(N = 155)	Typical carcinoid (N = 41)	Atypical carcinoid(N = 41)	Large cell neuroendocrine carcinoma (N = 41)	Small cell carcinoma(N = 32)	p-Value
Age, median (range)	67 (16–84)	67 (16–82)	64 (20–81)	69 (48–84)	70 (57–82)	0.05
Sex, N (%)						<0.001
Male	92 (59.4)	10 (24.4)	22 (53.7)	35 (85.4)	25 (78.1)	
Female	63 (40.6)	31 (75.6)	19 (46.3)	6 (14.6)	7 (21.9)	
Smoking status, N (%)						<0.001
Never	45 (29.0)	22 (53.7)	20 (48.8)	3 (7.3)	0 (0)	
Current	41 (26.5)	5 (12.2)	9 (22.0)	18 (43.9)	9 (28.1)	
Former	69 (44.5)	14 (34.1)	12 (29.2)	20 (48.8)	23 (71.9)	
Site of tumor, N (%)						<0.001
Peripheral	89 (57.4)	20 (48.8)	17 (41.5)	34 (82.9)	18 (56.3)	
Central	64 (41.3)	21 (51.2)	24 (58.5)	7 (17.1)	12 (37.5)	
Peripheral + central*	2 (0.7)	0 (0)	0 (0)	0 (0)	2 (6.2)	
Size of tumor (cm), median (range)	2.7 (0.5–15)	2.2 (0.7–8)	3 (0.8–8.5)	2.8 (0.5–15)	2.9 (1–9.5)	0.01
pT, N (%)						<0.001
T1	85 (54.8)	35 (85.3)	20 (48.8)	19 (46.3)	11 (34.4)	
T2	36 (23.2)	4 (9.8)	14 (34.1)	8 (19.6)	10 (31.2)	
T3–T4	34 (22.0)	2 (4.9)	7 (17.1)	14 (34.1)	11 (34.4)	
pN, N (%)						<0.001
N0	113 (72.9)	40 (97.6)	28 (68.3)	27 (65.8)	18 (56.3)	
N1	22 (14.2)	1 (2.4)	10 (24.4)	5 (12.2)	6 (18.7)	
N2	20 (12.9)	0 (0)	3 (7.3)	9 (22.0)	8 (25.0)	
pM, N (%)						0.21
M0	150 (96.8)	41 (100)	38 (92.7)	39 (95.1)	32 (100)	
M1	5 (3.2)	0 (0)	3 (7.3)	2 (4.9)	0 (0)	
Pleural involvement, N (%)						<0.001
Absent	120(77.4)	41 (100)	36 (87.8)	24 (58.5)	19 (59.4)	
Present	35 (22.6)	0 (0)	5 (12.2)	17 (41.5)	13 (40.6)	
Vascular invasion, N (%)						<0.001
Absent	126(81.3)	39 (95.1)	35 (85.4)	36 (87.8)	16 (50.0)	
Present	29 (18.7)	2 (4.9)	6 (14.6)	5 (12.2)	16 (50.0)	
Pathological AJCC stage, N (%)						<0.001
I	87 (56.1)	38 (92.7)	20 (48.8)	19 (46.3)	10 (31.2)	
II	35 (22.6)	2 (4.9)	14 (34.1)	8 (19.6)	11 (34.4)	
III	28 (18.1)	1 (2.4)	4 (9.8)	12 (29.2)	11 (34.4)	
IV	5 (3.2)	0 (0)	3 (7.3)	2 (4.9)	0 (0)	
Pattern of necrosis, N (%)						<0.001
Absent	66 (42.6)	41 (100)	24 (58.5)	0 (0)	1 (3.1)	
Punctate	31 (20.0)	0 (0)	17 (41.5)	5 (12.2)	9 (28.1)	
Extensive	39 (25.2)	0 (0)	0 (0)	24 (58.6)	15 (46.9)	
Geographic	19 (12.2)	0 (0)	0 (0)	12 (29.2)	7 (21.9)	
Growth patterns, N (%)						<0.001
Organoid	92 (59.4)	26 (63.4)	26 (63.4)	25 (61.0)	15 (46.9)	
Rosettes	11 (7.1)	3 (7.3)	7 (17.1)	1 (2.4)	0 (0)	
Spindle	16 (10.3)	2 (4.9)	3 (7.3)	0 (0)	11 (34.4)	
Trabecular	9 (5.8)	6 (14.6)	1 (2.4)	2 (4.9)	0 (0)	
Follicular	11 (7.1)	4 (9.8)	4 (9.8)	3 (7.3)	0 (0)	
Palisading	10 (6.5)	0 (0)	0 (0)	10 (24.4)	0 (0)	
Diffuse-lymphoma like	6 (3.8)	0 (0)	0 (0)	0 (0)	6 (18.7)	
TILs, N (%)						<0.001
None	44 (28.4)	24 (58.5)	19 (46.3)	1 (2.4)	0 (0)	
Focal	45 (29.0)	15 (36.6)	16 (39.1)	9 (22.0)	5 (15.6)	
Moderate	63 (40.6)	2 (4.9)	6 (14.6)	29 (70.7)	26 (81.3)	
Diffuse	3 (2.09	0 (0)	0 (0)	2 (4.9)	1 (3.1)	
Number of mitosis, median (range)	6 (0–90)	1 (0–1)	3 (2–9)	35 (17–90)	44 (25–78)	<0.001
Immunohistochemistry						
% Ki-67 median, (range)**	33 (1–95)	5 (1–15)	15 (5–45)	70 (40–90)	90 (50–95)	<0.001
Chromogranin A pos, N (%)**	146 (94.2)	41 (100)	41 (100)	36 (87.8)	28 (87.5)	<0.001
Synaptophysin pos, N (%)**	141 (91.0)	40 (97.6)	41 (100)	34 (82.9)	26 (81.3)	<0.001
CD56 pos, N (%)**	155 (100)	41 (100)	41 (100)	41 (100)	32 (100)	0.01
TTF-1 pos, N (%)**	71 (45.8)	0 (0)	2 (4.9)	38 (92.7)	31 (96.9)	<0.001

The table shows only the number of positive samples (intensity > 0).

TILs, tumor-infiltrating lymphocytes; pos, positive; AJCC, American Joint Committee on Cancer.

*Not considered for statistics.

**For the statistical studies, all immunohistochemical variables were considered as linear.

Patients with high-grade LCNEC and SCLC tumors were more frequently males and smokers. High-grade NETs were more often peripheral. SCLCs, LCNECs, and ACs were larger than TC tumors.

Patients with TC were more often pT1 without regional LN involvement.

Therefore, TC tumors were more commonly resected at stage I (N = 38; 92.7%) in contrast to other histotypes. As regards pleural involvement, high-grade NETs often presented invasion of the pleura, and SCLC showed more frequent vascular invasion than all the other NETs.

Necrosis presence and mitosis number were used as criteria to differentiate the different lung NETs (4). No TC tumors had necrosis, while some AC tumors had punctate necrosis (N = 17; 41.5%), and almost all high-grade NETs showed extensive or geographic necrosis. As expected, the Ki-67 index value was significantly higher in LCNECs and SCLCs compared with low-grade NETs.

With regard to architectural patterns, organoid was the most frequent pattern in low-grade NETs (TCs and ACs) and in LCNECs. The peripheral palisading pattern was observed only in LCNECs; similarly, the diffused lymphoma-like pattern was observed only in SCLCs. High-grade NETs presented significantly more TILs than low-grade tumors.

### DLL3 Immunohistochemistry in Lung NETs

**Figure 2:**
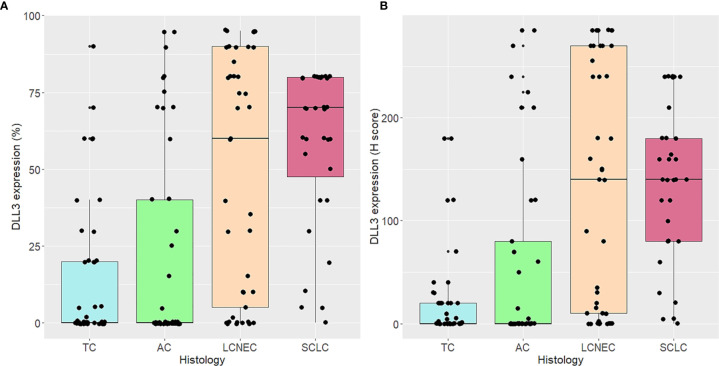
Delta-like protein 3 (DLL3) expression according to histological types. DLL3 expression is indicated as percentage of tumor cells **(A)** and H-score **(B)**.

DLL3 staining was positive in 99/155 (64%) samples, and high DLL3 expression was frequently observed in high-grade tumors. In particular, 20/41 (48.8%) LCNECs and 15/32 (46.9%) SCLCs showed a DLL3 H-score ≥150, whereas only 2/41 (4.9%) TCs and 8/41 (19.5%) ACs had an H-score as high as 150. Considering the percentage of tumor cells, 22/41 (53.7%) LCNECs, 24/32 (75%) SCLCs, 5/41 (12.2%) TCs, and 10/41 (24.4%) ACs showed more than 50% of stained tumor cells (**Figures 2A, B**). There were no significant differences in DLL3 expression within low-grade and high-grade tumors. Detailed DLL3 immunohistochemistry (IHC) results are shown in **Table 2**.

**Table 2:** Demographics and clinical and pathological features of all neuroendocrine tumors based on high DLL3 expression.

	DLL3 score %			DLL3 H-score		
	Low (<50%) 94/155	High (≥50%) 61/155	p-Value	Low (<150) 110/155	High (≥150) 45/155	p-Value
Histological diagnosis			<0.001			<0.001
TC, N (%)	36 (38.3)	5 (8.2)		39 (35.5)	2 (4.4)	
AC, N (%)	31 (33)	10 (16.4)		33 (29.9)	8 (17.8)	
LCNEC, N (%)	19 (20.2)	22 (36.1)		21 (19.1)	20 (44.5)	
SCLC, N (%)	8 (8.5)	24 (39.3)		17 (15.5)	15 (33.3)	
Age, median (range)	68 (16–84)	67 (40–79)	0.24	66 (16–84)	70 (40–79)	0.26
Male sex, N (%)	54 (57.4)	38 (62.3)	0.79	60 (54.6)	32 (71.1)	0.48
Smoking status, N (%)			<0.001			<0.001
Never	37 (39.4)	8 (13.1)		42 (38.1)	3 (6.7)	
Current	16 (17)	25 (41)		17 (15.5)	24 (53.3)	
Former	41 (43.6)	28 (45.9)		51 (46.4)	18 (40)	
Peripheral site of tumor, N (%)	41 (43.6)	48 (78.7)	<0.001	52 (47.3)	37 (82.2)	<0.001
Size of tumor (cm), median (range)	2.8 (0.7–9.5)	2.5 (0.5–15)	0.83	2.8 (0.7–9.5)	2.5 (0.5–15)	0.67
pT, N (%)			0.66			0.51
T1	55 (58.5)	30 (49.2)		61 (55.5)	24 (53.3)	
T2	21 (22.3)	15 (24.6)		27 (24.5)	9 (20)	
T3–T4	18 (19.2)	16 (26.2)		22 (20)	12 (26.7)	
pN, N (%)			0.20			0.27
N0	71 (75.5)	42 (68.9)		83 (75.5)	30 (66.7)	
N1	12 (12.8)	10 (16.4)		13 (11.8)	9 (20)	
N2	11 (11.7)	9 (14.7)		14 (12.7)	6 (13.3)	
pM1, N (%)	2 (2.1)	3 (4.9)	0.10	2 (1.8)	3 (6.7)	0.07
Pleural involvement, N (%)			<0.001			<0.001
Presence	12 (12.8)	23 (37.7)		17 (15.5)	18 (40)	
Absence	82 (87.2)	38 (62.3)		93 (84.5)	27 (60)	
Vascular invasion, N (%)	13 (13.8)	16 (26.2)	0.22	20 (18.2)	9 (20)	0.09
Pathological AJCC stage, N (%)			0.39			0.35
I	57 (60.6)	30 (49.2)		65 (59.1)	22 (48.9)	
II	19 (20.3)	16 (26.2)		23 (20.9)	12 (26.7)	
III	16 (17)	12 (19.7)		20 (18.2)	8 (17.8)	
IV	2 (2.1)	3 (4.9)		2 (1.8)	3 (6.6)	
Necrosis, median (range)	0 (0–60)	20 (0–60)	<0.001	0 (0–60)	20 (0–60)	<0.001
Pattern of necrosis, N (%)			<0.001			<0.001
Absent	56 (59.6)	10 (16.4)		62 (56.4)	4 (8.9)	
Punctate	13 (13.8)	18 (29.5)		18 (16.3)	13 (28.9)	
Extensive	17 (18.1)	22 (36.1)		20 (18.2)	19 (42.2)	
Geographic	8 (8.5)	11 (18)		10 (9.1)	9 (20)	
Growth patterns, N (%)			0.01			0.01
Organoid	57 (60.6)	35 (57.4)		66 (60)	26 (57.8)	
Rosettes	9 (9.6)	2 (3.3)		9 (8.2)	2 (4.4)	
Spindle	7 (7.4)	9 (14.8)		10 (9.1)	6 (13.3)	
Trabecular	8 (8.5)	1 (1.6)		9 (8.2)	0 (0)	
Follicular	9 (9.6)	2 (3.3)		10 (9.1)	1 (2.2)	
Palisading	3 (3.2)	7 (11.5)		3 (2.7)	7 (15.6)	
Diffuse-lymphoma like	1 (1.1)	5 (8.2)		3 (2.7)	3 (6.6)	
TILs, N (%)			<0.001			<0.001
Diffuse	0 (0)	3 (4.9)		1 (0.9)	2 (4.4)	
Moderate	26 (27.7)	37 (60.7)		34 (30.9)	29 (64.4)	
Focal	32 (34)	13 (21.3)		36 (32.7)	9 (20)	
None	36 (38.3)	8 (13.1)		39 (35.5)	5 (12.1)	
Number of mitosis, median (range)	3 (0–90)	35 (1–78)	<0.001	3 (0–90)	33 (1–78)	<0.001
Immunohistochemistry						
% Ki-67 median (range)*	10 (1–95)	80 (2–95)	<0.001	15 (1–95)	75 (2–95)	<0.001
Chromogranin A pos, N (%)*	85 (90.4)	51 (83.6)	0.27	97 (88.2)	39 (86.7)	0.40
Synaptophysin pos, N (%)*	87 (92.6)	54 (88.5)	0.24	101 (91.8)	40 (88.9)	0.20
CD56 pos, N (%)*	94 (100)	61 (100)	0.16	110 (100)	45 (100)	0.06
TTF-1 pos, N (%)*	28 (29.8)	43 (70.5)	<0.001	39 (35.5)	32 (71.1)	<0.001

The table shows only the number of positive samples (intensity > 0).

TILs, tumor-infiltrating lymphocytes; pos, positive; DLL3, delta-like protein 3.

TC, typical carcinoid; AC, atypical carcinoid; LCNEC, large cell neuroendocrine carcinoma; SCLC, small cell lung carcinoma; AJCC, American Joint Committee on Cancer.

*For the statistical studies, all immunohistochemical variables were considered as linear.

### DLL3 Immunohistochemistry and Clinicopathological Data

The association between DLL3 expression and clinicopathological patient characteristics and morphological findings is summarized in **Table 2**.

Overall, patients with high DLL3 expression were more frequently smokers, both current and former; high DLL3 expression was also associated with peripheral tumors.

Considering high- and low-grade tumors as separate groups (**Tables 3** and **4**), the high DLL3 expression was again associated with the peripheral site of the neoplasm (p = 0.01 for high-grade and p < 0.001 for low-grade tumors). In the high-grade neoplasm group, high DLL3 H-score was associated with advanced pathological American Joint Committee on Cancer (AJCC) stage and younger age.

**Table 3:** Demographics and clinical and pathological features of high-grade neuroendocrine tumors (73/155) based on high DLL3 expression.

	DLL3 score %			DLL3 H-score		
	Low (<50%) 27/73	High (≥50%) 46/73	p-Value	Low (<150) 38/73	High (≥150) 35/73	p-Value
Age, median (range)	73 (58–84)	67 (48–79)	<0.01	72 (58–84)	69 (48–79)	0.01
Male sex, N (%)	24 (88.9)	36 (78.3)	0.25	30 (78.9)	30 (85.7)	0.76
Smoking status, N (%)			0.12			0.06
Never	1 (3.7)	2 (4.3)		2 (5.3)	1 (2.8)	
Current	8 (29.6)	19 (41.3)		8 (21.1)	19 (54.3)	
Former	18 (66.7)	25 (54.3)		28 (73.7)	15 (42.9)	
Peripheral site of tumor, N (%)	16 (59.3)	36 (78.3)	0.05	23 (60.5)	29 (82.9)	0.01
Size of tumor (cm), median (range)	3.5 (1–9.5)	2.6 (0.5–15)	0.60	3.4 (1–9.5)	2.5 (0.5–15)	0.34
pT, N (%)			0.27			0.10
T1	10 (37.0)	20 (43.5)		13 (34.2)	17 (48.6)	
T2	8 (29.6)	10 (21.7)		12 (31.6)	6 (17.1)	
T3–T4	9 (33.4)	16 (34.8)		13 (34.2)	12 (34.3)	
pN, N (%)			0.61			0.23
N0	13 (48.1)	32 (69.6)		20 (52.6)	25 (71.4)	
N1	5 (18.5)	6 (13.0)		6 (15.8)	5 (14.3)	
N2	9 (33.4)	8 (17.4)		12 (31.6)	5 (14.3)	
pM1, N (%)	0 (0)	2 (4.3)	0.07	0 (0)	2 (5.7)	0.10
Pleural involvement, N (%)			0.93			0.60
Presence	10 (37.0)	20 (43.5)		15 (39.5)	15 (42.9)	
Absence	17 (63.0)	26 (56.5)		23 (60.5)	20 (57.1)	
Vascular invasion, N (%)	6 (22.2)	15 (32.6)	0.29	13 (34.2)	8 (22.9)	0.70
Pathological AJCC stage, N (%)			0.06			0.04
I	9 (33.3)	20 (43.5)		12 (31.6)	17 (48.6)	
II	7 (25.9)	12 (26.1)		11 (28.9)	8 (22.9)	
III	11 (40.7)	12 (26.1)		15 (39.5)	8 (22.9)	
IV	0 (0)	2 (4.3)		0 (0)	2 (5.7)	
Necrosis, median (range)	30 (5–60)	20 (0–60)	0.52	30 (0–60)	20 (10–60)	0.76
Pattern of necrosis, N (%)			0.74			1
Absent	0 (0)	1 (2.2)		1 (2.7)	0 (0)	
Punctate	2 (7.4)	12 (26.1)		7 (18.4)	7 (20.0)	
Extensive	17 (63.0)	22 (47.8)		20 (52.6)	19 (54.3)	
Geographic	8 (29.6)	11 (23.9)		10 (26.3)	9 (25.7)	
Growth patterns, N (%)			0.24			0.30
Organoid	17 (63.0)	23 (50.0)		22 (57.9)	18 (51.4)	
Rosettes	0 (0)	1 (2.2)		0 (0)	1 (2.8)	
Spindle	2 (7.4)	9 (19.6)		5 (13.1)	6 (17.1)	
Trabecular	1 (3.7)	1 (2.2)		2 (5.3)	0 (0)	
Follicular	3 (11.1)	0 (0)		3 (7.9)	0 (0)	
Palisading	3 (11.1)	7 (15.2)		3 (7.9)	7 (20.0)	
Diffuse-lymphoma like	1 (3.7)	5 (10.8)		3 (7.9)	3 (8.7)	
TILs, N (%)			0.12			0.28
Diffuse	0 (0)	3 (6.5)		1 (2.7)	2 (5.7)	
Moderate	19 (70.4)	36 (78.3)		27 (71.1)	28 (80.0)	
Focal	7 (25.9)	7 (15.2)		9 (23.5)	5 (14.3)	
None	1 (3.7)	0 (0)		1 (2.7)	0 (0)	
Number of mitosis, median (range)	35 (17–90)	40 (20–78)	0.60	38 (17–90)	39 (20–78)	0.67
Immunohistochemistry						
% Ki-67 median (range)*	80 (40–95)	80 (40–95)	0.30	80 (40–95)	80 (40–95)	0.28
Chromogranin A pos, N (%)*	18 (66.7)	36 (78.3)	0.07	25 (65.8)	29 (82.9)	0.04
Synaptophysin pos, N (%)*	21 (77.8)	39 (84.8)	0.44	30 (78.9)	30 (85.7)	0.53
CD56 pos, N (%)*	27 (100.0)	46 (100.0)	0.91	38 (100.0)	35 (100.0)	0.66
TTF-1 pos, N (%)*	26 (96.3)	43 (93.5)	0.17	37 (97.3)	32 (91.4)	0.08

The table shows only the number of positive samples (intensity > 0).

TILs, tumor-infiltrating lymphocytes; pos, positive; DLL3, delta-like protein 3; AJCC, American Joint Committee on Cancer.

*For the statistical studies, all immunohistochemical variables were considered as linear.

**Table 4:** Demographics and clinical and pathological features of low-grade neuroendocrine tumors (82/155) based on high DLL3 expression.

	DLL3 score %			DLL3 H-score		
	Low (<50%)67/82	High (≥50%) 15/82	p-Value	Low (<150)72/82	High (≥150) 10/82	p-Value
Age, median (range)	65 (16–82)	66 (40–79)	0.54	65 (16–82)	71 (40–79)	0.54
Female sex, N (%)	37 (55.2)	13 (86.7)	<0.001	42 (58.3)	8 (80)	<0.001
Smoking status, N (%)			0.06			0.07
Never	36 (53.7)	6 (40)		40 (55.6)	2 (20)	
Current	8 (11.9)	6 (40)		9 (12.5)	5 (50)	
Former	23 (34.3)	3 (20)		23 (31.9)	3 (30)	
Peripheral site of tumor, N (%)	25 (37.3)	12 (80)	<0.001	29 (40.3)	8 (80)	<0.001
Size of tumor (cm), median (range)	2.5 (0.7–8.5)	2.3 (0.9–4.4)	0.09	2.5 (0.7–8.8)	1.9 (0.9–4.4)	0.16
pT, N (%)			0.15			0.16
T1	45 (67.2)	10 (66.7)		48 (66.7)	7 (70)	
T2	13 (19.4)	5 (33.3)		15 (20.8)	3 (30)	
T3–T4	9 (13.4)	0 (0)		9 (12.5)	0 (0)	
pN, N (%)			0.89			0.89
N0	58 (86.6)	10 (66.7)		63 (87.5)	5 (50)	
N1	7 (10.4)	4 (26.7)		7 (9.7)	1 (10)	
N2	2 (3)	1 (6.7)		2 (2.8)	4 (40)	
pM1, N (%)	0 (0)	2 (13.3)	0.30	0 (0)	2 (20)	0.21
Pleural involvement, N (%)			0.01			<0.01
Presence	2 (3)	3 (20)		2 (2.8)	3 (30)	
Absence	65 (97)	12 (80)		70 (97.2)	7 (70)	
Vascular invasion, N (%)	7 (10.4)	1 (6.7)	0.82	7 (9.7)	1 (10)	0.88
Pathological AJCC stage, N (%)			0.38			0.32
I	48 (71.6)	10 (66.7)		53 (73.6)	5 (50)	
II	12 (17.9)	4 (26.7)		12 (16.7)	4 (40)	
III	5 (7.5)	0 (0)		5 (6.9)	0 (0)	
IV	2 (3)	1 (6.7)		2 (2.8)	1 (10)	
Necrosis %, median (range)	0 (0–30)	0 (0–5)	0.66	0 (0–30)	5 (0–5)	0.25
Pattern of necrosis, N (%)			0.01			<0.001
Absent	56 (83.6)	9 (60)		61 (84.7)	4 (40)	
Punctate	11 (16.4)	6 (40)		11 (15.3)	6 (60)	
Extensive	0 (0)	0 (0)		0 (0)	0 (0)	
Geographic	0 (0)	0 (0)		0 (0)	0 (0)	
Growth patterns, N (%)			0.64			0.63
Organoid	40 (59.7)	12 (80)		44 (61.1)	8 (80)	
Rosettes	9 (13.4)	1 (6.7)		9 (12.5)	1 (10)	
Spindle	5 (7.5)	0 (0)		5 (6.9)	0 (0)	
Trabecular	7 (10.4)	0 (0)		7 (9.7)	0 (0)	
Follicular	6 (9)	2 (13.3)		7 (9.7)	1 (10)	
Palisading	0 (0)	0 (0)		0 (0)	0 (0)	
Diffuse-lymphoma like	0 (0)	0 (0)		0 (0)	0 (0)	
TILs, N (%)			0.34			0.34
Diffuse	0 (0)	0 (0)		0 (0)	0 (0)	
Moderate	7 (10.4)	1 (6.7)		7 (9.7)	1 (10)	
Focal	25 (37.3)	6 (40)		27 (37.5)	4 (40)	
None	35 (52.2)	8 (53.3)		38 (90.5)	5 (50)	
Number of mitosis, median (range)	1 (0–9)	3 (1–8)	0.11	1 (0–9)	3 (1–8)	0.04
Immunohistochemistry						
% Ki-67 median (range)*	7 (1–45)	15 (2–80)	0.11	8 (1–45)	20 (2–75)	0.02
Chromogranin A pos, N (%)*	67 (100)	15 (100)	0.66	72 (100)	10 (100)	0.72
Synaptophysin pos, N (%)*	66 (98.5)	15 (100)	0.43	71 (98.6)	10 (100)	0.35
CD56 pos, N (%)*	67 (100)	15 (100)	0.16	72 (100)	10 (100)	0.10
TTF-1 pos, N (%)*	2 (3)	0 (0)	0.85	2 (2.8)	0 (0)	0.84

The table shows only the number of positive samples (intensity > 0).

TILs, tumor-infiltrating lymphocytes; pos, positive; DLL3, delta-like protein 3; AJCC, American Joint Committee on Cancer.

*For the statistical studies, all immunohistochemical variables were considered as linear.

In the low-grade neoplasm group, the DLL3 expression was higher in females (p < 0.001). The high DLL3 expression correlated with histological parameters typically associated with high-grade NETs such as high mitosis number (p < 0.001), Ki-67 index (p < 0.0001), and presence of necrosis (p < 0.001). These correlations hold true for the low-grade tumor group, while no significant associations were observed between the DLL3 expression and these variables in the high-grade group.

A greater DLL3 expression was also observed in tumors with visceral pleura infiltration, where 23/35 (65.7%) had ≥50% positive tumor cells and 18/35 (51.4%) had H-score ≥150. On the other hand, considering cases with no pleural involvement, 82/120 (68.3%) had <50% positive tumor cells and 93/120 (77.5%) had H-score <150. These findings were confirmed in the low-grade group of tumors.

The high DLL3 expression in all samples correlates with the presence of moderate or diffuse TIL infiltration (p < 0.001), palisading growth pattern (p = 0.01), and positive TTF-1 immunohistochemical staining (p < 0.001).

Among the neoplasms with high DLL3 expression, 40/61 (65.6% using percentage value) and 31/45 (68.9% using H-score) presented a moderate-to-severe inflammatory infiltrate.

In the high-grade neoplasm group (total = 73), DLL3 H-score positively correlated with chromogranin A expression (p = 0.04).

### DLL3 H-Score and Survival Data

**Figure 3:**
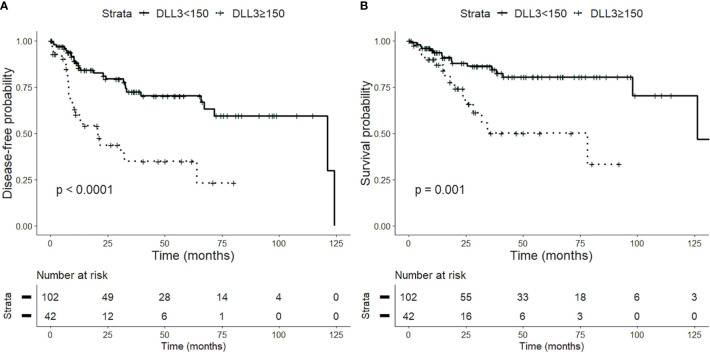
Impact of delta-like protein 3 (DLL3) expression on prognosis of patients with lung neuroendocrine tumors. High levels of DLL3 expression (H-score > 150) are associated with a worse disease-free survival (DFS) **(A)** and overall survival (OS) **(B)**.

**Figure 4:**
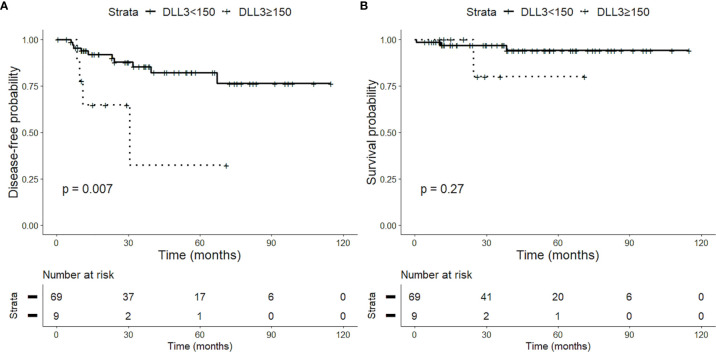
Impact of delta-like protein 3 (DLL3) staining on prognosis of patients with low-grade lung neuroendocrine tumors. High DLL3 expression (H-score > 150) is associated with a reduced disease-free survival (DFS) **(A)** but not with overall survival (OS) **(B)**, probably due to the low number of events.

The prognostic value of DLL3 was tested using an H-score cutoff of 150. Overall, the high DLL3 expression is associated with lower OS (p = 0.001) and DFS (p < 0.001) (**Figure 3**). Similarly, in low-grade tumors, the high DLL3 expression correlates with poorer DFS (p < 0.01) (**Figure 4**). As expected, the majority of adverse events in low-grade tumors occurred in AC patients. We tested the prognostic impact of DLL3 in this histological category, and again, the high DLL3 expression predicted a worse DFS (p = 0.01). To better understand the prognostic impact of DLL3, we tested it in multivariate settings, including histology and AJCC stage. DLL3 H-score (cutoff 150) showed a suggestive trend for poor DFS (p = 0.06, hazard ratio (HR) = 1.90, 95% CI 0.98–3.70), independently of the other parameters.

## Discussion

In the last few years, DLL3 has been identified as a novel therapeutic target gene mostly in SCLCs, but also in LCNECs (23, 36). Moreover, the literature data have demonstrated a relationship between DLL3 expression and sensitivity of platinum-based adjuvant chemotherapy, suggesting a predictive role of the DLL3 expression (37).

To the best of our knowledge, this is the first article investigating the DLL3 immunohistochemical expression and its prognostic role in a consecutive series of limited-stage lung NETs treated with surgery and including all four histological types (TCs, ACs, LCNECs, and SCLCs).

Higher DLL3 expression was more frequent in high-grade neoplasms. In detail, 46.9% and 75% of SCLC specimens showed a high DLL3 expression by using H-score and percentage of positive tumor cells, respectively. Our DLL3 prevalence data are consistent with those in the literature, showing that the DLL3 protein is highly expressed in SCLCs (21–23, 30, 35, 38, 39).

However, two studies reported a high DLL3 expression in only 32% of SCLCs (28). These discrepancies could depend on technical differences, on DLL3 score computation, and on the analysis of bioptic specimens.

We also demonstrated a high DLL3 expression in 48.8% of LCNECs by using H-score and 53.7% of LCNECs by using percentages of positive tumor cells, comparable with previous studies in LCNECs (29, 35). Only Ogawa and colleagues found lower rates of DLL3 expression (37.1% of LCNECs) by immunohistochemistry, but the results of their data could be due to the non-homogeneous study cohort, which included pure and combined LCNECs as well as different antibody clones (37).

The DLL3 expression has not been fully elucidated in lung carcinoid tumors. In our study, among the low-grade NETs, 20%–25% of ACs and 5%–12% of TCs—assessed by H-score and percentage of positive tumor cells, respectively—have high DLL3 expression. Only two other studies explored the DLL3 immunohistochemical expression in low-grade NETs. Alcala et al. reported a high expression in 40% of carcinoid samples (31); however, the authors included 20 low-grade NE neoplasms without specifying the TC and AC proportion, nor the DLL3 immunohistochemical expression cutoff used. The other study by Xie et al. showed higher DLL3 immunoreactivity in 37% of AC and 32.8% of TC samples by using the cutoff of >50% positive tumor cells to define the high DLL3 expression (30). However, we do not have a straightforward explanation for the DLL3 prevalence discrepancies observed in carcinoid cohorts. For this reason, the DLL3 expression in low-grade NETs needs to be evaluated in larger cohorts in order to define the possible prognostic-therapeutic role in this category of tumors.

The association between DLL3 expression and clinicopathological characteristics has not been thoroughly explored, and it is still largely uncertain. In our study, in the entire cohort, NE neoplasms with high DLL3 expression more often belonged to smoking patients. The neoplasms were mainly peripheral, and more than half of the high DLL3 expression neoplasms had pleural infiltration at microscopic evaluation. Samples with high immunoreactivity had higher numbers of mitosis, higher Ki-67 index, and greater necrosis; moreover, they generally presented palisade growth pattern and moderate-to-severe TILs and expressed TTF-1.

As regards high-grade NETs, those with high DLL3 expression tended to have advanced AJCC stage, peripheral location, and chromogranin A expression. The association of DLL3 expression with more aggressive tumor behavior was also found in patients with other high-grade tumor types, such as endometrial carcinoma, lung adenocarcinoma, and small cell bladder cancer (40–42), as well as in SCLC patients (39). However, other studies did not find any association between DLL3 expression and clinicopathological characteristics in high-grade NE lung tumors (28, 37, 38).

As regards low-grade NETs, neoplasms with high DLL3 expression frequently belonged to female patients, as previously described (30), and generally presented with a peripheral location. Interestingly, high DLL3 expression was also associated with aggressive histological characteristics, such as a higher number of mitoses, higher Ki-67 index, presence of punctate necrosis, and greater predisposition to pleural infiltration.

As regards survival data, high DLL3 expression was associated with lower OS and DFS in the entire cohort. The significant association with lower DFS was confirmed also independently of the histology and AJCC stage, which are the most useful prognostic indices. The association between high DLL3 expression and lower OS and DFS suggests that this marker might be associated with more aggressive tumors, even if this association has not been confirmed for high-grade tumors. The high proportion of patients with positive DLL3 tumor expression, despite the absence of prognostic implications, confirms previous results in SCLC and LCNEC patients (28, 37–39). A study by Huang et al. (43) found an association between high level of the DLL3 expression and low progression-free survival (PFS) and OS rates in biopsy from primary tumors and metastatic LNs in advanced SCLC patients. However, a larger multicenter study, evaluating DLL3 expression in biopsy samples collected from 1,073 SCLC patients with limited and extensive stage disease, did not find any association between DLL3 expression and survival data (32). Therefore, the DLL3 prognostic role needs to be further investigated in biopsy from SCLC patients, which represent the most frequent type of specimen in these patients. Only Xie and collaborators observed a significant association between high DLL3 expression and better OS and small size of tumors in both SCLC and AC patients, suggesting that the DLL3 expression might represent a favorable prognostic factor in lung NETs. However, in their study, only a relatively small percentage of lung NETs had low expression of DLL3. Therefore, prognostic data need to be interpreted with caution (30).

The DLL3 expression has not yet been associated with OS in low-grade NETs. We observed a significant association between high DLL3 expression and lower DFS in ACs. This finding as well as the association between DLL3 expression and aggressive histological characteristics suggests that DLL3 expression could identify a subgroup of ACs with worse prognosis and more clinically aggressive behavior.

Several recent studies suggest the existence of low-grade lung NETs with proliferative capacities higher than those currently accepted for TC and AC (5, 44, 45). These cases could be the lung equivalent of gastro-entero-pancreatic (GEP) NET G3. This new category has a prognostic and therapeutic significance: G3 NETs show a more aggressive behavior than G1–G2 NETs and a lower response rate to platinum-based chemotherapy, which remains a therapeutic signature of NE carcinomas (46). However, actually, this entity is not included in lung NET classification since only a limited number of cases have been reported so far, with different terminologies, different inclusion criteria, and few therapeutic information; and more data about these cases are needed. In this context, DLL3 expression could add useful prognostic information to histological subtyping in low-grade lung NETs.

Cardnell and collaborators found a correlation between TTF1 and DLL3 expression in SCLCs, suggesting that TTF1 could be used as a surrogate marker for DLL3 (47). However, we did not observe this association in high-grade tumors, which confirms previous results from the literature (29). In accordance with previous reports (29, 37), we observed a significant correlation between higher DLL3 expression and increased staining intensity of chromogranin A in high-grade tumors, which supported the hypothesis that the DLL3 expression is related to NE differentiation and could promote NE tumorigenesis in high-grade lung NETs.

Several limitations associated with the present study should be mentioned. Firstly, this was a retrospective, non-randomized single-center study that concerned only resected specimens. However, in clinical practice, the vast majority of SCLCs are diagnosed by biopsy procedure without any need for subsequent surgery resection. Nevertheless, we evaluated DLL3 expression by using a homogeneous cohort composed of only surgically resected pulmonary NETs. Our results thus need to be confirmed in a prospective cohort, by evaluating the DLL3 expression in biopsies from SCLC patients. Our cohort selection may also have led to a bias to evaluate the association between DLL3 expression and prognosis (OS and DFS) in SCLC patients, since the better outcome of the early-stage SCLC patients in our study. However, our results are similar to those of other studies that used biopsies of SCLC to assess for DLL3 expression (23, 32). Secondly, although our report is referred to a large series in terms of surgically resected SCLCs, the overall number of TCs, ACs, and LCNECs is relatively small; and further validation studies should be warranted.

However, despite these limitations, our study demonstrated a high prevalence of DLL3 expression in high-grade lung NET patients and its association with aggressive clinicopathological features. These findings confirm that DLL3 could represent a useful biomarker for target therapy in high-grade tumors. Our results also suggest that the DLL3 expression could identify a subset of ACs tumors with more aggressive behavior, thus providing the basis for new therapeutic options in this group of patients.

## Data Availability Statement

The original contributions presented in the study are included in the article/supplementary files. Further inquiries can be directed to the corresponding author.

## Ethics Statement

The study was approved by the Ethics Committee “Comitato Etico di Area Vasta Nord Ovest” (CEAVNO) for Clinical experimentation. Informed written consent for publication was not asked, since no data that can potentially and clearly identify patients were used and reported in the manuscript.

## Author Contributions

Conception of the work and result interpretation: GA, IDS, and GF. Immunohistochemistry and data analysis: AMP, SR, and AP. Sample collections and result interpretation: FD, ML, and FM. Pathological diagnosis: GA, IDS, and GF. All authors contributed to the article and approved the submitted version.

## Conflict of Interest

The authors declare that the research was conducted in the absence of any commercial or financial relationships that could be construed as a potential conflict of interest.

## Publisher’s Note

All claims expressed in this article are solely those of the authors and do not necessarily represent those of their affiliated organizations, or those of the publisher, the editors and the reviewers. Any product that may be evaluated in this article, or claim that may be made by its manufacturer, is not guaranteed or endorsed by the publisher.
